# Age-Specific Mortality During the 1918 Influenza Pandemic: Unravelling the Mystery of High Young Adult Mortality

**DOI:** 10.1371/journal.pone.0069586

**Published:** 2013-08-05

**Authors:** Alain Gagnon, Matthew S. Miller, Stacey A. Hallman, Robert Bourbeau, D. Ann Herring, David JD. Earn, Joaquín Madrenas

**Affiliations:** 1 Département de Démographie, Université de Montréal, Montreal, Quebec, Canada; 2 Department of Microbiology, Icahn School of Medicine at Mount Sinai, New York, United States of America; 3 Deparment of Sociology, Western University, London, Ontario, Canada; 4 Department of Anthropology, McMaster University, Hamilton, Ontario, Canada; 5 Department of Mathematics and Statistics, McMaster University, Hamilton, Ontario, Canada; 6 M.G. DeGroote Institute for Infectious Disease Research, McMaster University, Hamilton, Ontario, Canada; 7 Department of Microbiology and Immunology, McGill University, Montreal, Quebec, Canada; University of Edinburgh, United Kingdom

## Abstract

The worldwide spread of a novel influenza A (H1N1) virus in 2009 showed that influenza remains a significant health threat, even for individuals in the prime of life. This paper focuses on the unusually high young adult mortality observed during the Spanish flu pandemic of 1918. Using historical records from Canada and the U.S., we report a peak of mortality at the exact age of 28 during the pandemic and argue that this increased mortality resulted from an early life exposure to influenza during the previous Russian flu pandemic of 1889–90. We posit that in specific instances, development of immunological memory to an influenza virus strain in early life may lead to a dysregulated immune response to antigenically novel strains encountered in later life, thereby increasing the risk of death. Exposure during critical periods of development could also create holes in the T cell repertoire and impair fetal maturation in general, thereby increasing mortality from infectious diseases later in life. Knowledge of the age-pattern of susceptibility to mortality from influenza could improve crisis management during future influenza pandemics.

*“The war is over – and I must go”*

*Egon Schiele, 1890–1918.*

## Introduction

The atypically high mortality among young adults during the 1918 influenza pandemic remains unexplained and continues to trouble virologists and immunologists [1]. Few observers have examined the age-pattern of mortality in detail (but see references [2] and [3]). In this paper, we built from a forthcoming study [3] by analysing yearly ages at death during the fall wave of the 1918 pandemic in various locations in Canada and the USA and report a peak at the exact age of 28. Exploring the shape of the distribution of deaths leads us to propose immunological mechanisms that may help explain the atypically high young adult mortality in 1918, and relate that unusual pattern to prior exposure to the Russian influenza pandemic in 1889–90. Following the “original antigenic sin” [4], [5] or the “antigenic imprinting” [6] hypotheses, and adding insights from Shanks and Brundage [7] on T-cell dysregulation, we propose that development of immunological memory to a specific influenza strain early in life may dysregulate the immune response and thus increase the risk of death when encountering a novel and highly antigenically dissimilar strain in later life. This hypothesis is expanded to explain both the mortality peak at age 28 and the distribution around that peak in 1918. We first review current hypotheses regarding the atypical mortality pattern during the 1918 pandemic. Second, we present an analysis of mortality data gathered from historical sources and, third, we explore the immunological mechanisms that could account for the results.

## Common Hypotheses

The 1918 A (H1N1) Spanish flu pandemic was notable for being atypically fatal to those aged 20–40 years, a pattern widely noticed around the world [7]–[16]. The reasons for this observation are not clear. We list the four major theories:

1. The high proportion of young people who fell victim to the epidemic has been taken to imply that older people had acquired protective immunity from an earlier influenza outbreak with similar antigenic properties [4], [11], [17], [18]. Although useful, this explanatory scheme remains incomplete. “Antigenic history” [6] certainly aids in comprehending the low mortality among older people in 1918, but does not explain the high mortality rates among the young adults [6], [19]. Expanding on this, it has also been proposed that there is a “honeymoon period” of infectious diseases that occurs between the ages of 4 and 14 that protects younger individuals from morbidity and mortality [20]. While this likely played a role in protecting the young, it does not fully account for the sharp increase in mortality observed in individuals above 14 years of age.
2. According to numerous sources, mortality from tuberculosis increased among young male adults during World War I [21], raising the possibility that some of the increased mortality during the 1918 pandemic might be attributable to the deleterious consequences of concomitant tuberculosis and influenza infection [22]. This hypothesis rests on a higher male mortality during the 1918 influenza pandemic and therefore cannot be extended to other parts of the world where females died in greater proportion than males [23].
3. Another hypothesis suggests that the high mortality of young adults may be due to an overactive immune response (i.e., cytokine storm) at the height of immunocompetency [24]. Inoculating monkeys with the reconstructed 1918 influenza H1N1 virus, Kobasa et al. [25] attributed its unprecedented lethality to an “aberrant innate immune” response. Infected animals mounted a dysregulated antiviral response that was not only insufficient for protection but ultimately caused a highly pathogenic respiratory infection that killed them. This hypothesis fails to explain the unique age-specific trend of mortality observed during the 1918 influenza pandemic, and does not account for other factors likely important in determining outcome, chiefly, pre-exposure to earlier strains of influenza virus (immunological memory).
4. Finally, Shanks and Brundage recently identified T-cell dysregulation as the main culprit [7]. Historical records and animal models suggest that individuals exposed at least once to the (presumably) A/H3Nx 1889–90 pandemic strain were likely to have dysregulated cellular immune responses to infections with the A/H1N1 strain during the 1918 outbreak. This mechanism could also include the generation of antigenic peptides that act as T cell receptor antagonists on the anti-influenza response in the 1918 epidemic [26]. The immunopathologic effects might have increased susceptibility to lethal secondary bacterial pneumonia. This last explanation explicitly implicates exposure to the 1889–90 influenza pandemic. We argue that such a connection is fundamental to the understanding of the age pattern of mortality in 1918.

## The Russian Influenza of 1889–90

The Russian influenza virus caused one of the first epidemics tracked worldwide [27], [28]. Originating from the Eurasian Steppes, it spread through Russia to Western Europe during the fall of 1889 and arrived in the port cities of northeastern North America in late December 1889. By January the epidemic had crossed the Mid-West and entered Canada and by February/March 1890 it had spread to most regions of the continent. Newspapers of the time and other historical or contemporary sources [27], [29]–[31] attest to the presence of “La grippe” in all the locations studied in this paper (Table 1). Overall, the Russian flu was far less lethal than the Spanish flu but its clinical attack rate (proportion of people with clinical signs) of 30–60% was just as high, explaining its rapid global spread [27]. Diffusion was swift, leaving an impression of simultaneity. Vital registers show, for instance, a very sudden outburst of mortality in early January in Montreal, followed by a quick return to “normal” mortality after January 20. The disease returned in the spring of 1891 when new outbreaks were reported in New York City. However, no mention of the affliction was found for Canada that year and we were unable to detect through parish registers a second wave, even though illness from that virus could have been present in milder form. The 1890 pandemic is suspected to have been caused by an H3Nx influenza virus [7]. There is some North American serological evidence that it was similar to the H3N2 A/Hong Kong/1968 strain [32] and additional clues come from the observed lower mortality rates among the elderly (65+) during the H3 pandemic of 1968 [33].

**Table 1:** Source and number of deaths (from all causes) recorded by selected locations during the 1918 flu pandemic.

Location	Source of data	Number of deaths, October 1918 (all causes)	Number of deaths Sept-Dec 1918 (all causes)	Number of deaths Sept-Dec 1918 (flu)
Montreal (City)	Parish registers	3046	5366	N/A
Toronto (City)	Civil registers	1885	3071	2199
Hamilton (City)	Civil registers	175	962	542
Ottawa (City)	Civil registers	632	1100	640
London (City)	Civil registers	178	538	290
Welland & Lincoln (County)	Civil registers	319	907	550
Winnipeg (City)	Civil register indexes	216	1381	N/A
Vancouver (City)	Civil register indexes	532	1291	N/A
Philadelphia (City)	Civil registers, from [9]	14621	21780	13936
Indiana (State)	Civil registers, from [9]	5821	19270	9940
Kansas (State)	Civil registers, from [9]	3297	10983	5965

## Clues from Exact Age Distribution of Mortality

An alternative to the four explanations outlined above relies on the possibility that early life exposure to the 1889–90 influenza virus may have led to an *increased* susceptibility to a severe outcome following infection from the pandemic influenza virus of 1918 [3]. To investigate this, we analyzed registered death records from locations in Canada and published reports of mortality from the USA where appropriate data were available (see Table 1).

**Figure 1:**
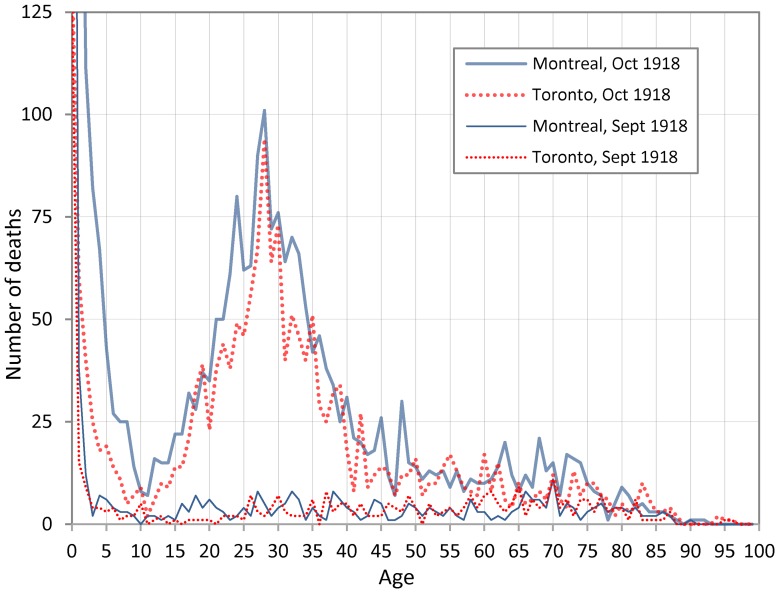
Recorded deaths (from all causes) by age in Montreal and Toronto, September and October, 1918.

Figure 1 presents the number of deaths by age recorded for October 1918 during the deadliest wave of the Spanish flu in Montreal and Toronto. We utilized microfilmed parish registers for Montreal and microfilmed death registrations for Toronto. The values for September 1918, the month preceding the pandemic, are also reported for comparison. The elevated number of deaths among young adults aged 20–40 that was noted in contemporary accounts and reinforced by subsequent analyses is evident. Figure 1 also shows a clear mode at age 28 for both cities. The peak at this age seen in [3] for Toronto is confirmed here for Montreal. Previous investigations have collapsed yearly ages at death into age-groups (20–24, 25–29, etc.) which masked mortality peaks at specific ages; however, Viboud et al. [2] also recently reported exact age-specific death rates for Kentucky in 1918 (see discussion).

As direct and reliable population totals are unavailable for 1918, it is quite difficult to calculate trustworthy rates of death by ages during the pandemic. In principle, it is possible to interpolate population totals by age from census data, but estimates obtained from these sources may be severely distorted by population processes such as the migrations that occurred in Canada after the end of the First World War. Census districts do not always match the administrative districts used in vital registration [34], leading to an additional source of bias. Further, historical data are often affected by age heaping, which occurs because age declarations are often rounded up or down to the nearest number that ends in 0, 2, 5, or 8. The level of such bias can be estimated with Myers' summary index [35], which measures the preference for a specific terminal digit (a value of 0 for that index represents no heaping while 90 indicates that all deaths are reported at the same terminal digit). In our data, the summary index for the death records for Canadian locations pooled together is 4.21. Heaping is also present in historical censuses and is usually more important for this type of records than for death records. In the 1921 Canadian census, the Myers index at ages 30–99 is 9.50 for Toronto and 8.06 for Montreal.

Despite the above difficulties and for the sake of completeness, we report in Figure et al 2 mortality rates for the cities of Montreal and Toronto during the month of October 1918. The denominators that were used to estimate these rates were based on the 1921 Canadian census. For Toronto, we found a population total for 1918 in the *Canada Year Book* [36], which was used to estimate a rate of growth during the 3 year interval; we applied this rate to each age in the 1921 census in order to obtain the population counts by age in 1918. For Montreal, we could not find a credible population total for 1918 but we found one for 1917 in the same *Canada Year Book*; the rate of increase between 1917 and 1921 was used instead to interpolate a total population size in 1918. Additionally, death counts from Montreal were taken from Catholic parish registers, which meant that we needed to estimate the proportion of Catholics living in the city during the pandemic in order to derive an appropriate denominator. For simplicity, we assumed that this proportion was the same in 1918 as it was in the 1921 census. We also report rates that buffer out fluctuations due to age heaping and small numbers using locally weighted regressions (lowess) [37]. For these rates, both the numerator and the denominator were smoothed prior to taking the ratio of the two.

**Figure 2:**
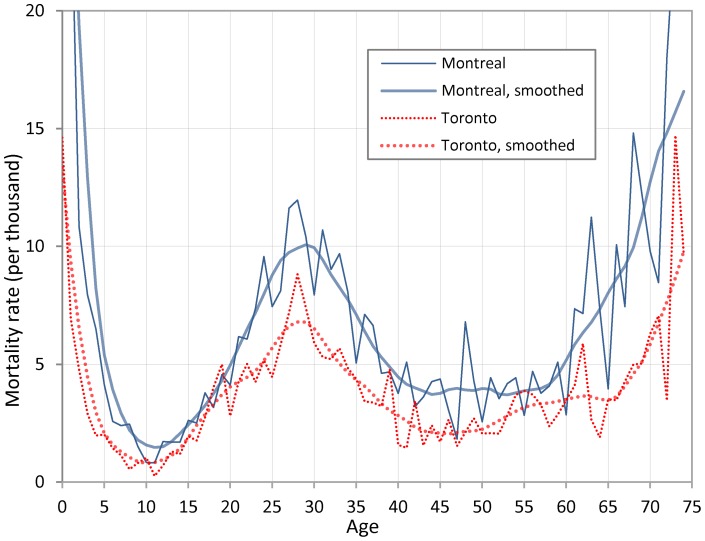
Death rates (from all causes) by age in Montreal and Toronto during the month of October, 1918.

As expected, using mortality rates instead of death counts does not fundamentally alter the results (Figure 2). Obviously, in relative terms, rates appear higher than death counts at older ages because there are fewer people alive at older ages (making the unsmoothed rates quite erratic and preventing us from plotting rates after age 75). Yet, revealing the well-known W-shaped curve of the Spanish influenza, mortality rates peak at approximately the same age (28–29) as death counts (28). The peak is less pronounced for the smoothed curves but this mostly depends on the bandwidth of the lowess. Using a shorter bandwidth (which amounts to a smaller sliding window for a moving average), we obtain a sharper high point, centered at age 28. It is difficult, however, to know to what extent excess mortality at age 28 is genuine or results from a particular attraction for that age. Further, it is important to realize that the years surrounding the 1918 pandemic included two major disruptions to the population pyramid: the First World War and the pandemic itself, which certainly introduces biases, especially in the estimation of the population at risk. The migrations or the delocalization of young individuals and young families that followed the end of the First World War make the estimation even more difficult and extreme caution should be exercised in its interpretation.

We now introduce additional locations in the study in order to see whether the pattern noticed for Montreal and Toronto can be generalized. Since death rates reported for the USA by the Bureau of the Census [9] also show evidence of notable estimation problems, we decided to leave aside these rates and to extract the raw death counts instead. For the same reason, we refrained from using age-specific population numbers from Canadian census data in the remainder of this paper.

**Figure 3:**
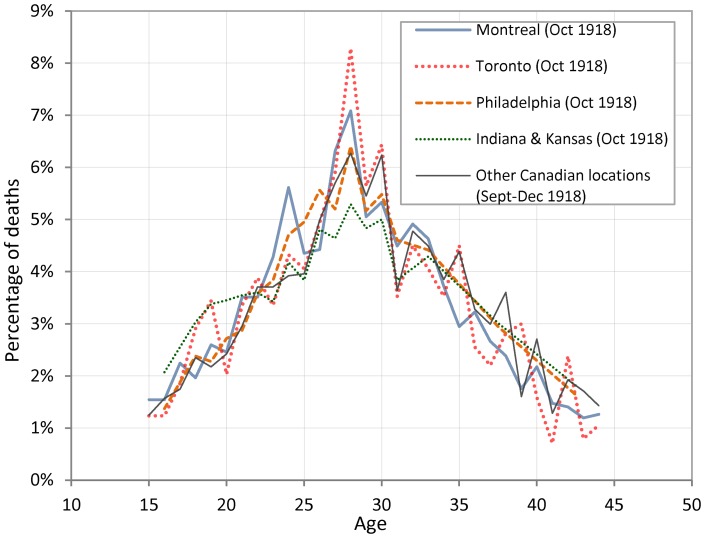
Distribution of deaths (from all causes) by age as a percentage of all deaths between ages 15 and 44 in all available Canadian and American locations. ^*^ *Key for **Figure 3:** Yearly age-specific death counts were available from age 18 to 31 for Philadelphia, Indiana, and Kansas in the special tables that were tabulated for these locations in 1920 (27); Outside this range, death counts were only available for collapsed age-groups (i.e., 15–17, 32–34, 35–39, and 40–44). For these age-groups we divided the number of deaths in the interval by its length and plotted the obtained number at the midpoint value of the interval. Other Canadian locations: Hamilton, Ottawa, London, Welland & Lincoln, Winnipeg, and Vancouver.

Figure 3 reports the distribution of deaths between ages 15 and 45 as a percentage of all deaths within this age-range. We utilized the same data for Montreal and Toronto as in Figure 1 and added other data from death registrations from Ontario and death registration indexes from other Canadian locations, as well as death counts reported in the Bureau of the Census tabulations for locations in the US [9]. We used percentages to facilitate comparisons and pooled deaths from some locations from September to December to prevent random fluctuations and overcrowding of the figure. The distributions are remarkably similar. A mode at age 28 is evident, with a secondary mode at age 30 in some cases. What is seen for fewer Canadian locations in [3] is confirmed for American locations. The patterns are relatively similar for males and females (not shown here). The exceptions are mostly due to the absence of significant numbers of young adult males in some cases (especially in the U.S. locations, where deaths from soldiers, sailors and marines were not counted in [9]), which leads to a smaller number of male deaths at younger ages in comparison with female deaths. Yet, in Philadelphia, death counts peak at age 28 for both males and females. On the other hand, in Indiana and Kansas, the modes were off by a unit or two when analyses were based on males or females separately. When males and females for both States were pooled, the peak was again at age 28, as reported in Figure 3. There are also similar discrepancies at the local level in the Canadian data that vanish when data are pooled together.

**Figure 4:**
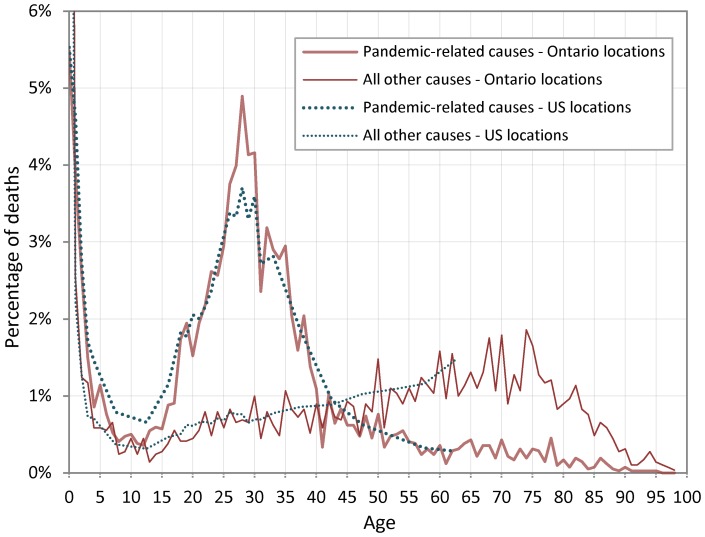
Percentages of deaths by age from pandemic-related causes and from all other causes in Ontario and US locations, September to December 1918. **^&&^Key for** **Figure 4:** Yearly age-specific deaths counts were available from age 0 to 4 and from 18 to 31 for the US locations in the special tables that were tabulated for these locations in 1920 (27); outside these ranges, death counts were only available for collapsed age-groups (i.e., 5–9, 10–14, 15–17, 32–34, 35–39, 40–44, ..., and 60–64). We divided the number of deaths in the interval by its length and plotted the obtained number at the midpoint value of the interval. Deaths above age 65 were not available by age or age-groups for the U.S. locations. Ontario locations: Toronto, Hamilton, Ottawa, London, and Welland & Lincoln; US locations: Philadelphia, Indiana, and Kansas.

Finally, we use additional information on causes of death, which was available only for the three American and the five Ontarian locations. In order to estimate mortality caused by the pandemic, deaths attributed to influenza and pneumonia and to influenza, pneumonia, and bronchitis were extracted from the US tabulations [9] and from the Ontario registers, respectively. In Figure 4, densities are reported for pandemic-related mortality and for mortality from all other causes from September to December 1918. For instance, of all the individuals whose death certificate indicated a flu death during those months in the Ontario data, 4.9% were 28 years old. The corresponding figure for non-flu death at the same age is 0.7%. The percentages for the US locations are very similar, except for higher fractions of flu deaths between ages 5 and 15 (with corresponding lower fractions between ages 25 and 30) and a less marked peak at age 28. For other causes of death, the two distributions are practically identical.

None of the four models outlined above can fully account for the peculiar age distribution of mortality reported here. Supposing that a H1N1-like virus circulated before the 1889 pandemic, multiple exposures to drifting variants of this virus prior to 1889 could account for the decrease in mortality for those above age 28 in 1918 (i.e., the “antigenic history” model). However, it is hard to imagine how people aged 28 that year would have had less protective immunity from earlier circulation of H1N1influenza viruses than their younger counterparts. Similarly, the other three explanatory schemes would not lead to the prediction that mortality should peak at a precise age in a number of places separated by thousands of kilometers. Although explanatory scheme (4) comes close to this prediction by explicitly referring to earlier infection with the 1889–90 pandemic strain, it does not predict higher mortality for any specific age: in this scheme, previous exposure alone, whatever the age, would have sufficed to trigger T-cell dysregulation in 1918.

Close inspection of Figures 1, 2, 3 and 4 suggests alternative views. The general form of age-specific mortality data in 1918, with a peak at a specific age, suggests that individuals were more or less *susceptible* to dying from influenza given their age at the time of the 1889–90 pandemic, rather than being more or less *protected* from earlier exposure to an antigenically similar virus. Since mortality peaks at age 28 in 1918, it seems straightforward to suppose that exposure during development and/or very early in life to the 1889–90 strain led to more severe response to infection in 1918. We now propose a few plausible immunological scenarios that are consistent with this profile.

## Subverting the Immune Response: Antigenic Imprinting

The phenomenon of “original antigenic sin” (OAS) was first described in the early 1950s by Thomas Francis and colleagues [4]. Their analysis of serum samples from field studies of influenza infections revealed minimal immunological responses against the current viral strain but a response instead directed towards a strain previously encountered as children. Numerous studies have found further evidence of OAS [5], [6], [25], [38], [39]. One mechanistic explanation for this is that conserved, but non-neutralizing epitopes on the secondary viruses elicit a memory antibody response generated during the first infection that is faster and greater in magnitude than the *de novo* response, but not protective against the new strain. As a result, these memory cells essentially out-compete the protective cells that would normally be newly generated against the subsequent exposures.

According to Reichart et al. [5], OAS is a possible explanation for the case age distribution during the recent 2009 flu pandemic, which was biased toward younger individuals [40], as in 1918. Indeed, one salient feature of the novel H1N1 in 2009 was that it lacked glycosylation sites on the globular head of the hemagglutinin, a pattern also shared with the 1918 pandemic strain and H1N1 viruses that circulated until the early 1940s. Exposure to progressively more drifted (and glycosylated) H1N1 seasonal strains in successive cohorts would have produced an immune response increasingly mismatched to the novel 2009 H1 in progressively younger peoples.

Such subversion of immunity may have also taken place in 1918 for people born before 1890. If an H1N1-like virus were circulating and drifting during the decades prior to the 1889–90 antigenic shift, then it is possible that those born around 1888 were exposed early in life to a strain that was antigenically farther from the 1918 strain than those born in 1878. If so, the susceptibility to severe outcome and mortality from influenza among those aged 30 would have been higher in the 1918 pandemic than for those aged 40. This “antigenic seniority” [39] could explain the decrease of mortality at older ages from its peak at age 28 during the 1918 pandemic. Older individuals would also have been more “immunologically experienced” with H1N1 viruses from a greater number of re-exposures. This too may have resulted in progressively lower mortality in those 30 years and older at the time of the 1918 pandemic. We now turn to discuss the increase in mortality up to age 28.

Strictly speaking, OAS refers to the tendency of the immune system to use immunological memory based on a previous infection when a subsequent, slightly different version of that virus is encountered. Being “trapped” by the first response the immune system is unable to build up more effective responses during subsequent exposures. However, in the context of influenza viruses the expression is most often employed to refer more specifically to the predominant production of antibodies to the first influenza strain encountered. To avoid confusion, it may be useful to see OAS as a special case of “antigenic imprinting” [6], a more general immunological mechanism that would include all instances of “commitment” to the first-exposure strain.

Our model of antigenic imprinting may be explained as an imbalance between the cellular and humoral branches of the adaptive immune response [41], [42]. As written above, the 1890 pandemic is suspected to have been caused by an H3Nx influenza virus. Despite a probable lack of cross-protective antibodies between H1N1 and H3Nx, these two subtypes would almost certainly have shared many T cell epitopes [43]. The model would then predict that a first encounter with the 1889–90 H3Nx virus early in life generates robust cytotoxic T cell memory that could have been recalled upon later exposure to the 1918 pandemic H1N1 virus. However, without the complement of protective antibodies, the uncoupled cellular immune response may have gone unchecked, resulting in severe immunopathology of the lung [44] and death. Those born later in the 1890 decade and who thus first “committed” early in life to progressively drifted strains of this virus may have had progressively decreased severity due to: a decrease in shared cytotoxic T cell epitopes on the internal viral proteins [45]; a decrease in virulence of the 1890 virus as it drifted [5], [46]; and/or improved herd immunity over subsequent years of drift that resulted in decreased rates and severity of illness [47]. All of these would decrease the magnitude of the cytotoxic T cell memory and thus the potential of detrimental immunopathology upon infection in 1918. Thus, the risk of mortality in 1918 would have been progressively lower for people born later in the 1890 s, especially for those individuals between ages 14–27, no longer in the “honeymoon period.”

## Other Mechanisms: Exposure during Critical Periods of Development

The neonatal immune system continues to develop until approximately 6 months of age [48]. Prior to this, neonates are incapable of mounting normal immune responses to infection and rely on maternal antibodies. Thus, according to our antigenic imprinting hypothesis, people born a year or so before 1890 were at a higher risk of death during the 1918 pandemic because they first encountered (and developed an immune response to) the H3Nx strain at the youngest possible age. This would heighten death tolls at a slightly older age than age 28, say 29 or 30. Yet, the death count peaks exactly at age 28 during the fall of 1918, i.e., presumably for individuals who were less than 6 months old at the time of the Russian flu pandemic (peaking in January 1890 in North America [27]). In this context, it could be alternatively proposed that exposure to influenza during early development *in utero* or in infancy in 1890 resulted in permanent alteration of immune function that indirectly led to increased mortality during the subsequent 1918 outbreak. For instance, deletion or anergy of specific T cell clones due to influenza exposure during thymic development could have created “holes” in the T cell repertoire with an associated increased risk of death later in life from infectious diseases [49], [50]. In this “critical period” framework, the earlier the insult, the more severe the long term effect. That is, clonal deletion *in utero* may have been more substantial than in infancy [51], thereby heightening mortality at age 28 during the 1918 pandemic.

Exposure to influenza early in life could also affect later life mortality through other pathways. Historical studies of epidemics often report increased adult mortality and morbidity among those who were born at a time of an epidemic. Almond [52] found that exposure to the 1918 influenza virus in late stages of fetal development was associated with higher adult cardiovascular disease prevalence later in the 20^th^ century. Following infection, the maternal immune response may divert resources from the growing fetus and, like nutritional deprivation, affect fetal maturation with permanent changes in glucose-insulin metabolism and later life implications for cardiovascular health (c.f. the Barker and thrifty phenotype hypotheses [53], [54]). Since the last trimester of gestation is important for lung maturation [55], exposure during this critical period of development may also increase adult respiratory disease mortality. Recently, Myrskylä et al. [56] found that exposure in the last trimester of pregnancy during the 1918 flu pandemic led to increased mortality risks from cardiovascular and respiratory diseases later in life. High infectious disease load during the first year of life could also cause irreversible damage to health. Two studies in England demonstrated that exposure to airborne infectious diseases at that age are associated with cough, phlegm, and impaired ventilatory function [57]. In 18^th^–19^th^ century Sweden, individuals born during years of smallpox and whooping cough epidemics had an increased risk of death after age 50 [58], [59].

## Discussion

Emergence of virulent influenza viruses through antigenic shift and drift remains a significant threat to public health [18]. The next pandemic will emerge in unpredictable form and have unforeseen consequences for the age pattern of morbidity and mortality. Although immunological studies are of primary importance, historical data provide important information about the context in which a virulent strain of influenza originates and sweeps through populations.

In understanding the unusual age-specific morbidity and mortality that occurred during the 1918 influenza virus pandemic, it is important to distinguish between intrinsic susceptibility to infection, and susceptibility to severe outcomes following infection. Intrinsic age-specific susceptibility to influenza virus infection in 1918 has been difficult to assess due to the inability to de-convolute multiple confounding factors, including pre-existing immunity or conditions arising from the First World War. However, no convincing data exists to suggest any major age-specific variations in intrinsic susceptibility to influenza virus infection except in pediatric and elderly populations. Therefore, it is highly unlikely that such differences could account for the pronounced differences in morbidity and mortality of 28 year olds versus those who were 40–60 during 1918.

We propose that the major antecedent of the Spanish flu pandemic was the “Russian flu” pandemic and that the time distance between the two is crucial for understanding age-specific mortality in 1918. We argue that developing immunological memory to an antigenically dissimilar influenza subtype in early life may actually subvert the immune system, thereby increasing the risk of death when the individual is infected by a novel strain in later life. As explained above, such a mechanism would elucidate the atypical shape of mortality during the 1918 pandemic reported here. Exposure during a critical period of development (*in utero* or in infancy) could also permanently affect later life health and mortality through clonal changes in the T cell repertoire, impaired lung maturation or metabolism alterations. Yet, despite compelling evidence, these explanations remain incomplete. At a minimum, more detailed analyses using exact ages derived from historical birth and death records for 1889–90 and 1918 are needed.

A major difficulty with the data at hand is indeed the possibility of age heaping, which as we noted earlier may distort historical data counts. This could have affected the results reported by Viboud et al. [2], who found a peak of mortality at age 26 for Kentucky during the fall of 1918, and not at age 28. Age heaping in the 1910 and 1920 American censuses does not seem to have been accounted for in that paper. Population estimates for 1918 were obtained by interpolating between the two censuses. This perhaps inflated the denominators used to calculate the death rate at age 28, given that people that age in 1918 were 20 and 30 in 1910 and 1920 respectively (age heaping is highest for numbers with zero as a terminal digit). Myers indexes, which calculate the extent of heaping, were fairly high in U.S. censuses for the first decades of the 20^th^ century [35], [60]. Smoothing techniques can be used to flatten an age distribution affected by age heaping, but it is not clear whether interpolating population numbers between two censuses separated by 10 years can provide reliable exact age estimates [60], especially since this 10 year interval included major disturbances to the population pyramid as the First World War and the 1918 influenza pandemic. Ma et al. [6] who also used data from US censuses for a preliminary analysis on age-specific mortality risk from influenza pandemic, found a mode in mortality rates at ages 30–34 for the country as a whole, corresponding to a peak age at least 4–5 years older than the peak of 26 found by Viboud et al. [2] for Kentucky. Ma et al. [6] renounced from using population sizes and to calculate mortality rates in their finer grain analysis of yearly age-specific mortality during the 1957 and 1968 pandemics in Canada.

In this study, we also avoided the distortions associated with early 20^th^ century censuses by focusing on age-specific death counts, not rates, even though we reported rates in order to illustrate that our findings do not disappear when rates are calculated. This resulted in a peak in influenza deaths centered on a single age –28– over a wide range of geographic locations. It is certainly possible that death counts themselves are influenced by age-heaping at ages 28 and 30. However, redistribution using locally weighted regression in Figure 2 was not large enough to significantly affect the pattern.

Ma et al. [6] found that years with unusually large antigenic changes (1918, 1928, and 1946) delineated boundaries for birth years (ages) with increased mortality during the 1957 pandemic, in contrast with our observation that exposure early in life to the previous 1889–90 pandemic was associated with peak mortality in 1918. Although antigenic imprinting appears relevant in both cases, it seems to have led in one instance to differential protective immunity (in 1957) and in the other to differential severity (1918), likely because of the sequential ordering of the pandemic hemagglutinin subtypes. The globular head domains of H1 and H2 exhibit substantial antigenic differences, however, the stalk domain on which they rest is nearly identical [61], likely inducing a substantial level of cross-protective immunity between the two subtypes [unpublished data]. The H3 hemagglutinin, however, differs in both head and stalk domains from H1 and H2 subtypes, and therefore unlikely elicited a cross-protective response to either of the two. If the 1890 pandemic was indeed caused by a H3Nx subtype, commitment very early in life to this subtype would have led to an immune profile offering little or no humoral protection to the antigenically dissimilar H1N1 subtype that emerged in 1918, leaving the pattern of age-specific mortality dominated by the differential severity described in this paper. Finally, the age-specific expression of morbidity and mortality in any pandemic is contingent on the population's experience of previous pandemics.
